# 3-Methyl-*N*-(2-methyl­phen­yl)benzamide

**DOI:** 10.1107/S1600536810024578

**Published:** 2010-06-26

**Authors:** B. Thimme Gowda, Miroslav Tokarčík, Vinola Z. Rodrigues, Jozef Kožíšek, Hartmut Fuess

**Affiliations:** a Department of Chemistry, Mangalore University, Mangalagangotri-574 199, Mangalore, India; b Faculty of Chemical and Food Technology, Slovak Technical University, Radlinského 9, SK-812 37 Bratislava, Slovak Republic; c Institute of Materials Science, Darmstadt University of Technology, Petersenstrasse 23, D-64287, Darmstadt, Germany

## Abstract

The mol­ecular structure of the title compound, C_15_H_15_NO, involves an intra­molecular C—H⋯O hydrogen bond. The central amide group –NH—C(=O)– is twisted by 37.95 (12)° out of the *meta*-substituted benzoyl ring and by 37.88 (12)° out of the *ortho*-substituted aniline ring. The two benzene rings are inclined to one another at only 4.2 (1)° having an inter­planar spacing of *ca* 0.90 Å. The crystal structure is stabilized by inter­molecular N—H⋯O hydrogen bonds, which link the mol­ecules into chains running along the *b* axis. A weak inter­molecular C—H⋯π inter­action is also present.

## Related literature

For the preparation of the title compound, see: Gowda *et al.* (2003 ▶). For related structures, see: Bowes *et al.* (2003 ▶); Gowda *et al.* (2008**a* ▶,b* ▶); Rodrigues *et al.* (2010 ▶).
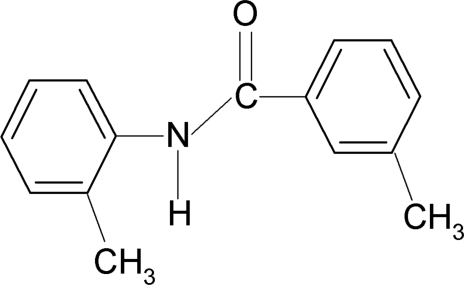


## Experimental

### 

#### Crystal data

C_15_H_15_NO
*M* *_r_* = 225.28
Monoclinic, 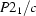

*a* = 11.1896 (3) Å
*b* = 4.95027 (14) Å
*c* = 24.1164 (5) Å
β = 116.512 (2)°
*V* = 1195.37 (5) Å^3^
*Z* = 4
Mo *K*α radiation
μ = 0.08 mm^−1^
*T* = 295 K
0.55 × 0.13 × 0.08 mm

#### Data collection

Oxford Diffraction Gemini R CCD diffractometer
Absorption correction: multi-scan (*CrysAlis PRO*; Oxford Diffraction, 2009 ▶) *T*_min_ = 0.954, *T*_max_ = 0.993
13694 measured reflections
2124 independent reflections
1553 reflections with *I* > 2σ(*I*)
*R*_int_ = 0.036

#### Refinement

*R*[*F* ^2^ > 2σ(*F* ^2^)] = 0.042
*wR*(*F* ^2^) = 0.126
*S* = 1.02
2124 reflections
156 parameters
1 restraint
H-atom parameters constrained
Δρ_max_ = 0.18 e Å^−3^
Δρ_min_ = −0.16 e Å^−3^

### 

Data collection: *CrysAlis PRO* (Oxford Diffraction, 2009 ▶); cell refinement: *CrysAlis PRO*; data reduction: *CrysAlis PRO*; program(s) used to solve structure: *SHELXS97* (Sheldrick, 2008 ▶); program(s) used to refine structure: *SHELXL97* (Sheldrick, 2008 ▶); molecular graphics: *ORTEP-3* (Farrugia, 1997 ▶) and *DIAMOND* (Brandenburg, 2002 ▶); software used to prepare material for publication: *SHELXL97*, *PLATON* (Spek, 2009 ▶) and *WinGX* (Farrugia, 1999 ▶).

## Supplementary Material

Crystal structure: contains datablocks I, global. DOI: 10.1107/S1600536810024578/vm2033sup1.cif

Structure factors: contains datablocks I. DOI: 10.1107/S1600536810024578/vm2033Isup2.hkl

Additional supplementary materials:  crystallographic information; 3D view; checkCIF report

## Figures and Tables

**Table 1:** Hydrogen-bond geometry (Å, °) *Cg*1 is the centroid of the C1–C6 ring.

*D*—H⋯*A*	*D*—H	H⋯*A*	*D*⋯*A*	*D*—H⋯*A*
N1—H1*N*⋯O1^i^	0.86	2.13	2.9417 (14)	157
C13—H13⋯O1	0.93	2.48	2.908 (2)	108
C14—H14c⋯*Cg*1^i^	0.96	2.70	3.627 (2)	161

Symmetry code: (i) 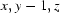.

**Fig. 1.**
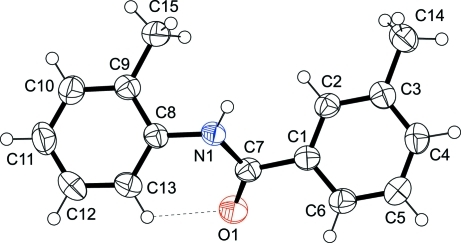
Molecular structure of (I) showing the atom labelling scheme and intramolecular C—H···O hydrogen bond (dashed line). Displacement ellipsoids are drawn at the 50% probability level and H atoms are represented as small spheres of arbitrary radii.

**Fig. 2.**
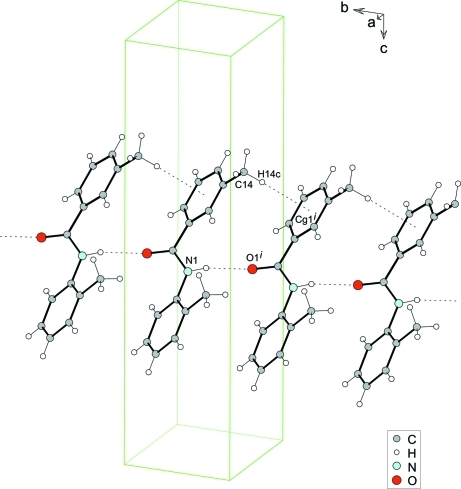
Part of the crystal structure of (I) showing the formation of a chain along [0 1 0] generated by N–H···O hydrogen bond. Another interaction within a chain is a weak C—H···π (arene) hydrogen bond involving the C14 methyl group and the centroid Cg1(i) of the C1—C6 ring. Hydrogen bonds are indicated by dashed lines. Symmetry code (i): x, y - 1, z.

## supplementary crystallographic information

### Comment

As part of a study of the substituent effects on the crystal structures of benzanilides (Bowes *et al.*, 2003; Gowda *et al.*, 2008*a,b*; Rodrigues *et al.*, 2010), in the present work, the structure of *N*-(2-methylphenyl)-3-methylbenzamide (I) has been determined (Fig. 1). In the crystal, the *ortho*-methyl substituent on the anilino ring is positioned *syn* to the N–H bond, while the *meta*-methyl substituent on the benzoyl ring is positioned *anti* to the carbonyl C==O bond.

The structure of (I) involves an intramolecular C–H···O hydrogen bond (Table 1) with the ring atom C13 as a donor and the amido O atom as an acceptor. The two benzene rings are inclined to one another at only 4.2 (1)° with an interplanar spacing of *ca*0.90 Å. The central amide group –NH–C(=O)- is twisted by 37.95 (12)° out of the *meta*-substituted benzoyl ring and by 37.88 (12)° of the *ortho*-substituted anilino ring. The crystal packing (Fig. 2) is dominated by intermolecular N–H···O hydrogen bonds which link the molecules into the chains along [0 1 0]. A weak intermolecular C—H···π(arene) hydrogen bond is also present in the structure, and occurs between the C14 methyl group and the centroid *Cg*1(i) of the C1—C6 ring at the position (i): *x*, *y* - 1, *z* (Table 1).

### Experimental

The title compound was prepared according to the method described by Gowda *et al.* (2003). The purity of the compound was checked by determining its melting point. It was characterized by recording its infrared and NMR spectra. Rod-like colourless single crystals of the title compound were obtained by slow evaporation from an ethanol solution of the compound (0.5 g in about 30 ml of ethanol) at room temperature.

### Refinement

All H atoms were visible in difference maps and then treated as riding atoms with C—H = 0.93 or 0.96 Å, N—H = 0.86 Å and O—H = 0.90 Å. The *U*_iso_(H) values were set at 1.2*U*_eq_(C aromatic, N) and 1.5*U*_eq_(C methyl, O). The *U* values of the bonded atoms C7 and O1  have been subject (using the DELU instruction) to a rigid bond restraint, thus enforcing their anisotropic displacement components in the direction of  the bond to be equal within a standard deviation of 0.005.

### Crystal data

**Table:**

C_15_H_15_NO	*F*(000) = 480
*M**_r_* = 225.28	*D*_x_ = 1.252 Mg m^−^^3^
Monoclinic, *P*2_1_/*c*	Mo *K*α radiation, λ = 0.71073 Å
Hall symbol: -P 2ybc	Cell parameters from 6151 reflections
*a* = 11.1896 (3) Å	θ = 3.4–29.4°
*b* = 4.95027 (14) Å	µ = 0.08 mm^−^^1^
*c* = 24.1164 (5) Å	*T* = 295 K
β = 116.512 (2)°	Rod, colorless
*V* = 1195.37 (5) Å^3^	0.55 × 0.13 × 0.08 mm
*Z* = 4	

### Data collection

**Table:**

Oxford Diffraction Gemini R CCD diffractometer	2124 independent reflections
graphite	1553 reflections with *I* > 2σ(*I*)
Detector resolution: 10.434 pixels mm^-1^	*R*_int_ = 0.036
ω scans	θ_max_ = 25.1°, θ_min_ = 2.1°
Absorption correction: multi-scan (*CrysAlis PRO*; Oxford Diffraction, 2009)	*h* = −13→13
*T*_min_ = 0.954, *T*_max_ = 0.993	*k* = −5→5
13694 measured reflections	*l* = −28→28

### Refinement

**Table:**

Refinement on *F*^2^	Primary atom site location: structure-invariant direct methods
Least-squares matrix: full	Secondary atom site location: difference Fourier map
*R*[*F*^2^ > 2σ(*F*^2^)] = 0.042	Hydrogen site location: inferred from neighbouring sites
*wR*(*F*^2^) = 0.126	H-atom parameters constrained
*S* = 1.02	*w* = 1/[σ^2^(*F*_o_^2^) + (0.085*P*)^2^] where *P* = (*F*_o_^2^ + 2*F*_c_^2^)/3
2124 reflections	(Δ/σ)_max_ < 0.001
156 parameters	Δρ_max_ = 0.18 e Å^−^^3^
1 restraint	Δρ_min_ = −0.16 e Å^−^^3^

### Special details

**Table:**

Geometry. All e.s.d.'s (except the e.s.d. in the dihedral angle between two l.s. planes) are estimated using the full covariance matrix. The cell e.s.d.'s are taken into account individually in the estimation of e.s.d.'s in distances, angles and torsion angles; correlations between e.s.d.'s in cell parameters are only used when they are defined by crystal symmetry. An approximate (isotropic) treatment of cell e.s.d.'s is used for estimating e.s.d.'s involving l.s. planes.
Refinement. Refinement of *F*^2^ against ALL reflections. The weighted *R*-factor *wR* and goodness of fit *S* are based on *F*^2^, conventional *R*-factors *R* are based on *F*, with *F* set to zero for negative *F*^2^. The threshold expression of *F*^2^ > σ(*F*^2^) is used only for calculating *R*-factors(gt) *etc*. and is not relevant to the choice of reflections for refinement. *R*-factors based on *F*^2^ are statistically about twice as large as those based on *F*, and *R*- factors based on ALL data will be even larger.

### Fractional atomic coordinates and isotropic or equivalent isotropic displacement parameters (Å^2^)

**Table:**

	*x*	*y*	*z*	*U*_iso_*/*U*_eq_	
C1	0.60977 (14)	0.5878 (3)	0.45102 (7)	0.0380 (4)	
C2	0.62846 (15)	0.3929 (3)	0.41406 (7)	0.0400 (4)	
H2	0.7127	0.3169	0.4269	0.048*	
C3	0.52474 (15)	0.3091 (3)	0.35868 (7)	0.0421 (4)	
C4	0.40013 (16)	0.4216 (3)	0.34128 (8)	0.0485 (4)	
H4	0.3288	0.3659	0.3046	0.058*	
C5	0.37946 (16)	0.6155 (3)	0.37739 (8)	0.0496 (4)	
H5	0.2947	0.6884	0.3649	0.06*	
C6	0.48384 (15)	0.7010 (3)	0.43174 (7)	0.0445 (4)	
H6	0.4701	0.8342	0.4555	0.053*	
C7	0.72176 (14)	0.6863 (3)	0.50986 (7)	0.0386 (4)	
C8	0.92671 (14)	0.5441 (3)	0.60094 (7)	0.0366 (4)	
C9	1.04382 (15)	0.4018 (3)	0.61249 (7)	0.0398 (4)	
C10	1.15481 (17)	0.4475 (3)	0.66806 (8)	0.0529 (5)	
H10	1.233	0.353	0.6767	0.064*	
C11	1.15358 (18)	0.6281 (4)	0.71109 (8)	0.0587 (5)	
H11	1.2302	0.6558	0.748	0.07*	
C12	1.03848 (18)	0.7672 (3)	0.69924 (8)	0.0543 (5)	
H12	1.0372	0.89	0.7282	0.065*	
C13	0.92508 (16)	0.7255 (3)	0.64467 (7)	0.0453 (4)	
H13	0.8471	0.8189	0.637	0.054*	
C14	0.54820 (18)	0.1002 (3)	0.31908 (8)	0.0551 (5)	
H14A	0.6314	0.1368	0.318	0.083*	
H14B	0.4767	0.107	0.2778	0.083*	
H14C	0.5514	−0.0761	0.3363	0.083*	
C15	1.04971 (16)	0.2057 (3)	0.56617 (8)	0.0499 (4)	
H15A	1.0146	0.2898	0.5261	0.075*	
H15B	0.9975	0.0487	0.5641	0.075*	
H15C	1.1407	0.1533	0.5787	0.075*	
N1	0.81081 (12)	0.4968 (2)	0.54447 (6)	0.0394 (3)	
H1N	0.796	0.3332	0.5311	0.047*	
O1	0.73096 (11)	0.92511 (18)	0.52470 (5)	0.0555 (4)	

### Atomic displacement parameters (Å^2^)

**Table:**

	*U*^11^	*U*^22^	*U*^33^	*U*^12^	*U*^13^	*U*^23^
C1	0.0434 (9)	0.0277 (7)	0.0421 (9)	0.0000 (6)	0.0184 (7)	0.0039 (6)
C2	0.0405 (9)	0.0322 (8)	0.0451 (9)	0.0024 (6)	0.0172 (8)	0.0041 (6)
C3	0.0503 (10)	0.0331 (8)	0.0414 (9)	−0.0044 (7)	0.0190 (8)	0.0033 (7)
C4	0.0452 (10)	0.0472 (9)	0.0439 (10)	−0.0068 (7)	0.0116 (8)	0.0023 (7)
C5	0.0404 (9)	0.0523 (10)	0.0530 (10)	0.0054 (7)	0.0180 (8)	0.0063 (8)
C6	0.0477 (10)	0.0391 (8)	0.0476 (9)	0.0036 (7)	0.0222 (8)	0.0013 (7)
C7	0.0415 (9)	0.0288 (8)	0.0462 (9)	−0.0012 (6)	0.0202 (7)	0.0015 (6)
C8	0.0420 (9)	0.0272 (7)	0.0388 (8)	−0.0049 (6)	0.0164 (7)	−0.0007 (6)
C9	0.0437 (9)	0.0309 (8)	0.0450 (9)	−0.0030 (6)	0.0199 (8)	−0.0003 (6)
C10	0.0439 (9)	0.0513 (10)	0.0548 (11)	0.0004 (7)	0.0141 (8)	−0.0028 (8)
C11	0.0535 (11)	0.0618 (11)	0.0454 (10)	−0.0098 (9)	0.0084 (9)	−0.0104 (9)
C12	0.0686 (12)	0.0491 (10)	0.0450 (10)	−0.0097 (8)	0.0252 (9)	−0.0135 (8)
C13	0.0505 (10)	0.0387 (8)	0.0486 (10)	−0.0018 (7)	0.0237 (8)	−0.0057 (7)
C14	0.0655 (12)	0.0476 (10)	0.0492 (10)	−0.0023 (8)	0.0228 (9)	−0.0065 (8)
C15	0.0497 (10)	0.0452 (9)	0.0545 (10)	0.0031 (7)	0.0230 (8)	−0.0056 (8)
N1	0.0435 (8)	0.0259 (6)	0.0423 (7)	−0.0005 (5)	0.0134 (6)	−0.0039 (5)
O1	0.0611 (8)	0.0243 (6)	0.0653 (8)	0.0002 (5)	0.0141 (6)	−0.0038 (5)

### Geometric parameters (Å, °)

**Table:**

C1—C6	1.390 (2)	C9—C10	1.380 (2)
C1—C2	1.391 (2)	C9—C15	1.504 (2)
C1—C7	1.495 (2)	C10—C11	1.374 (2)
C2—C3	1.385 (2)	C10—H10	0.93
C2—H2	0.93	C11—C12	1.373 (2)
C3—C4	1.381 (2)	C11—H11	0.93
C3—C14	1.508 (2)	C12—C13	1.376 (2)
C4—C5	1.383 (2)	C12—H12	0.93
C4—H4	0.93	C13—H13	0.93
C5—C6	1.376 (2)	C14—H14A	0.96
C5—H5	0.93	C14—H14B	0.96
C6—H6	0.93	C14—H14C	0.96
C7—O1	1.2262 (16)	C15—H15A	0.96
C7—N1	1.3517 (18)	C15—H15B	0.96
C8—C13	1.391 (2)	C15—H15C	0.96
C8—C9	1.402 (2)	N1—H1N	0.86
C8—N1	1.4194 (19)		
			
C6—C1—C2	119.03 (14)	C11—C10—C9	122.13 (16)
C6—C1—C7	118.77 (13)	C11—C10—H10	118.9
C2—C1—C7	122.16 (13)	C9—C10—H10	118.9
C3—C2—C1	121.63 (14)	C12—C11—C10	119.58 (16)
C3—C2—H2	119.2	C12—C11—H11	120.2
C1—C2—H2	119.2	C10—C11—H11	120.2
C4—C3—C2	118.03 (14)	C11—C12—C13	120.16 (15)
C4—C3—C14	121.44 (14)	C11—C12—H12	119.9
C2—C3—C14	120.54 (14)	C13—C12—H12	119.9
C3—C4—C5	121.22 (15)	C12—C13—C8	120.21 (15)
C3—C4—H4	119.4	C12—C13—H13	119.9
C5—C4—H4	119.4	C8—C13—H13	119.9
C6—C5—C4	120.25 (15)	C3—C14—H14A	109.5
C6—C5—H5	119.9	C3—C14—H14B	109.5
C4—C5—H5	119.9	H14A—C14—H14B	109.5
C5—C6—C1	119.82 (15)	C3—C14—H14C	109.5
C5—C6—H6	120.1	H14A—C14—H14C	109.5
C1—C6—H6	120.1	H14B—C14—H14C	109.5
O1—C7—N1	123.11 (14)	C9—C15—H15A	109.5
O1—C7—C1	121.14 (13)	C9—C15—H15B	109.5
N1—C7—C1	115.75 (12)	H15A—C15—H15B	109.5
C13—C8—C9	120.07 (14)	C9—C15—H15C	109.5
C13—C8—N1	121.22 (13)	H15A—C15—H15C	109.5
C9—C8—N1	118.71 (13)	H15B—C15—H15C	109.5
C10—C9—C8	117.84 (14)	C7—N1—C8	125.73 (12)
C10—C9—C15	120.56 (14)	C7—N1—H1N	117.1
C8—C9—C15	121.60 (14)	C8—N1—H1N	117.1
			
C6—C1—C2—C3	0.0 (2)	N1—C8—C9—C10	−179.00 (13)
C7—C1—C2—C3	−178.03 (13)	C13—C8—C9—C15	−179.54 (14)
C1—C2—C3—C4	−1.1 (2)	N1—C8—C9—C15	1.1 (2)
C1—C2—C3—C14	179.15 (13)	C8—C9—C10—C11	−0.8 (2)
C2—C3—C4—C5	1.1 (2)	C15—C9—C10—C11	179.06 (15)
C14—C3—C4—C5	−179.21 (15)	C9—C10—C11—C12	0.6 (3)
C3—C4—C5—C6	0.2 (2)	C10—C11—C12—C13	0.1 (3)
C4—C5—C6—C1	−1.4 (2)	C11—C12—C13—C8	−0.6 (2)
C2—C1—C6—C5	1.3 (2)	C9—C8—C13—C12	0.4 (2)
C7—C1—C6—C5	179.36 (14)	N1—C8—C13—C12	179.69 (14)
C6—C1—C7—O1	−36.9 (2)	O1—C7—N1—C8	−1.3 (2)
C2—C1—C7—O1	141.04 (15)	C1—C7—N1—C8	178.10 (13)
C6—C1—C7—N1	143.59 (14)	C13—C8—N1—C7	39.2 (2)
C2—C1—C7—N1	−38.4 (2)	C9—C8—N1—C7	−141.47 (15)
C13—C8—C9—C10	0.4 (2)		

### Hydrogen-bond geometry (Å, °)

**Table:**

Cg1 is the centroid of the C1–C6 ring.

**Table:**

*D*—H···*A*	*D*—H	H···*A*	*D*···*A*	*D*—H···*A*
N1—H1N···O1^i^	0.86	2.13	2.9417 (14)	157
C13—H13···O1	0.93	2.48	2.908 (2)	108
C14—H14c···Cg1^i^	0.96	2.70	3.627 (2)	161

Symmetry codes: (i) *x*, *y*−1, *z*.
